# 1014. Case Study. Skin Cell Suspension (SCSA) for Complex Necrotizing Soft Tissue Infections: A Case Series

**DOI:** 10.1093/jbcr/irag033.202

**Published:** 2026-04-06

**Authors:** Beverly Trutter, Marc Matthews, Lourdes Castañon

**Affiliations:** University of Arizona-Department of Surgery, Tucson, AZ; University of Arizona, Tucson, AZ; University of Arizona, Tucson, AZ

## Abstract

**Patient Presentation (age range, injury details, relevant history):**

Four females and 1 male with a median age of 62 with significant comorbidities who underwent reconstruction following extensive debridements.

**Clinical Challenges:**

Large surgical wounds following multiple debridements.

Intensive Care Unit Admission.

One patient in the cohort had Mucor.

**Management Approach:**

Multiple excisions and debridements.

Negative Pressure Wound Therapy in some of the cases.

Simultaneous Use of a Dermal Matrix, Split Thickness Skin Graft and Skin Cell Suspension Autograft.

**Outcomes:**

Skin Cell Suspension Autograft expands a small harvested donor site to cover large wounds.

Reduced donor site morbidity while promoting epithelialization and graft take.

Accelerated wound healing and reduced time to closure.

Decreased number of Operating Room Visits.

**Lessons Learned:**

reliable wound closure with high graft take.

Absence of donor site morbidity.

No major complications.

Closure achieved in 3-6 weeks with preserved limb salvage.

